# Fire and Rhizosphere Effects on Bacterial Co-Occurrence Patterns

**DOI:** 10.3390/microorganisms11030790

**Published:** 2023-03-19

**Authors:** Effimia M. Papatheodorou, Spiros Papakostas, George P. Stamou

**Affiliations:** 1 Department of Ecology, School of Biology, AUTH, 54124 Thessaloniki, Greece; 2 Department of Science and Technology, School of Science and Technology, University Center of International Programmes of Studies, International Hellenic University, 57001 Thessaloniki, Greece

**Keywords:** modularity, small-worldness, stochasticity, determinism, functional groups, network analysis

## Abstract

Fires are common in Mediterranean soils and constitute an important driver of their evolution. Although fire effects on vegetation dynamics are widely studied, their influence on the assembly rules of soil prokaryotes in a small-scale environment has attracted limited attention. In the present study, we reanalyzed the data from Aponte et al. (2022) to test whether the direct and/or indirect effects of fire are reflected in the network of relationships among soil prokaryotes in a Chilean sclerophyllous ecosystem. We focused on bacterial (genus and species level) co-occurrence patterns in the rhizospheres and bulk soils in burned and unburned plots. Four soils were considered: bulk-burnt (BB), bulk-unburnt (BU), rhizosphere-burnt (RB), and rhizosphere-unburnt (RU). The largest differences in network parameters were recorded between RU and BB soils, while RB and BU networks exhibited similar values. The network in the BB soil was the most compact and centralized, while the RU network was the least connected, with no central nodes. The robustness of bacterial communities was enhanced in burnt soils, but this was more pronounced in BB soil. The mechanisms mainly responsible for bacterial community structure were stochastic in all soils, whether burnt or unburnt; however, communities in RB were much more stochastic than in RU.

## 1. Introduction

Mediterranean climate is found in regions surrounding the Mediterranean Sea, the western coast of California, central Chile, southwestern South Africa, and southwestern Australia. Annual precipitation ranges from 275 to 950 mm, with over 65% falling during the six coldest months [1]. The above-ground vegetation in Mediterranean Type Ecosystems (MTΕs) is predominantly flammable evergreen sclerophyll shrubs and is considered a result of climate-induced ecological convergence [2]. Soils in MTΕs are shallow with a distorted soil profile, scattered surface rocks, and massive blocks of the outcropping parent rock. They are comparatively rich in nitrogen, poor in organic matter, and almost lacking in extractable phosphorus. The role of soil microbial assemblages in maintaining soil quality, vegetation productivity, and resilience against disturbances is crucial, given their close association with the root system [3,4,5].

In MTEs, overgrazing and fire are major drivers of ecological convergence [6,7]. Fire has been a tool used by humans to alter their environment since ancient times [8]. In addition to removing vegetation cover, fire causes serious changes in porosity and water retention capacity, electrical conductivity, soil organic matter, soil chemistry, and stoichiometry [9,10,11]. The combustion of vegetation results in the production of charcoal, a form of recalcitrant pyrogenic carbon [12,13,14]. The addition of charcoal to the soil causes pronounced changes in its physicochemical and biological characteristics. Particularly improving are the effects on soil moisture, porosity, pH, bulk density, organic C, and serving as an effective adsorbent for organic pollutants [15,16]. Charcoal persists in the soil for an extended period and has a long-term impact on soil microbial community structure and function [15]. It is notable that charcoal often creates a kind of ecotone between the soil and itself, known as the “charosphere” [12]. In short, charcoal is thought to function as fire’s black legacy [14].

Fire frequency and severity are key factors directly affecting plant and soil responses [17,18]. Post-fire regeneration in MTEs is a shrub encroachment process, as it starts with a prelude of grass and herbaceous vegetation that is gradually replaced by woody shrubs [19]. The duration of encroachment is variable. Arianoutsou-Faraggitaki [20] reported that in a Greek phryganic formation, the percentage contributions of woody and herbaceous species were almost the same as before the fire after seven years. In Chilean sclerophyll formations, encroachment is faster, taking only four years [21]. Post-fire regeneration in MTEs is achieved either by plant regeneration or by dormant seeds [22]. However, Chilean sclerophyll vegetation lacks soil seed banks, requiring regeneration through resprouting from buried bulbs, lignotubers, or roots and rarely by seeds dispersed by birds [23].

Although the complete recovery of soil quality requires many years [24,25], the recovery period for soil communities can range from months to three years [26,27,28]. The available literature has duly documented the post-fire, short-term reduction of soil microbial richness, biomass, and activity [11,26,28,29]. Key adaptive mechanisms of microbial recolonization include the ability of microbes to survive high temperatures combined with rapid growth rates as well as their ability to exploit new resources/niches created by fire [30]. Specifically, Pérez Valera et al. [31] suggested that changes in soil conditions during post-fire recovery may increase soil microbiota diversity and decrease vegetation diversity. Indeed, in MTEs worldwide, fire selectively favors plant species with seed persistence, reducing plant phylogenetic diversity, but the effect on microbiome dynamics remains contingent on soil resources and competition hierarchies [31], thereby reducing or increasing diversity. In this study, we recapitulated the data by Aponte et al. [27] referring to bacterial communities’ beta diversity and composition on phylum level in soils attached (rhizosphere) and detached (bulk) to plant roots in unburned and burned plots 33 months after a fire in a sclerophyllous formation close to Pumaque (central Chile). We reanalyzed the data to test whether the direct or indirect effects of fire are reflected in the network of relationships among prokaryotes. Our analysis focused on genus and species levels to investigate the microbial interactions at the lowest taxonomic level. Further, although the effect of fire on the mechanisms controlling the assembly of the soil bacterial community has been examined in some papers recently [14,32,33], to the best of our knowledge, no studies have focused on microbial co-occurrence patterns in small-scale environments, such as those of rhizospheres and bulk soils in burned and unburned plots.

Recently, the debate on the forces that govern the composition and structure of ecological communities has focused on the concepts of determinism and stochasticity [34]. Determinism refers to phylogenetically grounded processes that constitute a species’ niche. Dispersal limitation and species sorting through biotic and abiotic environmental filtering are argued to be the main deterministic forces [35,36]. Stochasticity refers to processes that involve randomness. Ecological drift resulting from stochastic changes in species abundance and unlimited dispersal is argued to be the main stochastic force [37]. Deterministic and stochastic processes are not mutually exclusive. Net deterministic and net stochastic perspectives occupy the two endpoints of a determinism-stochasticity continuum reflecting competitive and stochastic exclusion, respectively [38]. In the real world, the relative influence of determinism and stochasticity can vary in both time and space between the two endpoints of the continuum [36,39]. Discussion of the forces governing the composition and structure of ecological communities could also be informed by differences in the competitive ability and/or species’ niches. Mayfield and Levine [40] reformulated the ‘competition-relatedness’ or ‘limited similarity’ hypothesis, whereby phylogenetically closely related taxa compete more strongly than their distant counterparts, limiting their ability to coexist. They proposed that coexistence in competitive communities is driven by the interplay of two types of species differences, with a predominance of niche differences resulting in coexistence and a dominance of competition ability leading to over-dispersion of species. However, in the real world, species combine traits related to both niche and competitive ability differences. Intuitively, if niche differences are greater than differences in competitive ability, competitors will tend to coexist in a single cluster. Conversely, if differences in competitive ability are greater than differences in niches, species may be over-dispersed across multiple clusters.

Co-occurrence network analysis has recently gained popularity in analyzing biota relationships [41]. Since current studies have demonstrated that divergent bacterial communities are developed on the rhizosphere and the surrounding bulk soil [42], data sets from rhizospheres and bulk soils in burnt and unburnt plots were retrieved separately. Several network metrics are used to reveal mechanisms related to community assembly [39,41]. Modularity is key in exploring the determinism-stochasticity balance. Modular architectures are characterized by weakly interconnected subnets (modules) but strongly connected nodes within the subnet [43]. The connectivity pattern of nodes within individual modules is rather uniform and modulated by deterministic processes (processes in the niche realm). By contrast, the pattern of node connectivity at the scale of the entire network is rather randomly modulated by stochastic forces such as random oscillations in climate variables and unlimited dispersal of bacteria through wind and/or precipitation [40]. In this paper, the contribution of deterministic and stochastic processes in shaping the network architecture was determined using the Shannon Information Index to measure the uncertainty (entropy) contained in the relationships between nodes [44]. Finally, the assessment of network robustness was based on the consideration of their connectivity as measured by centrality metrics [45].

Specifically, the hypotheses tested were the following: (a) the accumulated dead material along with the fire-produced charcoal is key in shaping network architecture, (b) the contribution of deterministic and stochastic processes in shaping networks’ architectures, as well as network robustness varies across small spatial scales (rhizosphere vs. bulk soil), and (c) the stochastic control is expected to prevail in burnt soils since stochasticity is enhanced during the early post-fire period.

## 2. Materials and Methods

The analyzed data in this study were obtained from a prior study conducted by Aponte et al. [27] in the Pumanque commune (34° 350 44.9900 S; 71° 420 17.34900 W). The study site is located at an elevation of 100 m on the east front of the coastal mountain range and was affected by a forest fire event in 2017. The samples used in this study were collected 33 months later and were taken from a sclerophyllous Mediterranean forest with an understory consisting of 30 herbaceous species. To obtain the soil samples, three experimental plots of 20 × 20 m were marked in both burned and unburned areas (0.7 hectares each), with a distance of 40 to 60 m between their centroids. Within each experimental plot, nine equidistant points were marked using a 5 × 5 m grid to obtain the soil samples. A radial buffer area of 1 m was selected at each sampling point and was excavated using a shovel up to rooting deep (5–10 cm). This process allowed us to obtain rhizosphere soil samples by handshaking and collecting soil attached to roots of herbaceous species, as well as bulk soil samples from soil not attached to plant roots. Rhizosphere and bulk soil samples were pooled from each of the nine sampling points per plot to obtain six composite samples for bulk soil and six composite samples for rhizosphere in total. A detailed description of the study site and sampling procedures can be found in Aponte et al. [27].

Soil DNA was isolated using the DNeasy PowerSoil DNA isolation kit (QIAGEN, Valencia, CA, USA) according to the manufacturer’s instructions. Bacteria and Archaea were identified using the prokaryotic primer set 515f (5′-GTG YCA GCM GCC GCG GTA A-3′; [46]) and 806r (5′-GGA CTA CNV GGG TWT CTA AT-3′; [47]), which amplify a part of the V4 region of the 16S rRNA genes. Barcoded amplicon sequencing was performed on an Illumina MiSeq PE300 platform (Illumina, San Diego, CA, USA) at the All Genetics & Biology SL laboratory service facility (A Coruña, Spain) [27]. The raw sequencing reads were deposited in the Sequence Read Archive (SRA) of NCBI under accession number PRJNA784510, and the taxonomic distribution of reads that were analyzed in this study was obtained as processed by its SRA Taxonomy Analysis Tool (STAT) at NCBI. This tool has been shown to be an accurate and scalable framework that describes submitted microbial data [48].

We analyzed OTUs co-occurrence patterns in bulk-burnt (BB), bulk-unburnt (BU), rhizosphere-burnt (RB), and rhizosphere-unburnt (RU) soils by the CoNet software [49], which is part of the Cytospace 3.9.1. software [50]. Co-presence was identified using an ensemble-based network approach [32], which captures links between two measures of dissimilarity (Bray–Curtis and Kullback–Leibler) and one measure of similarity (mutual information) to reduce the impact of choosing a single measure [49]. Links detected by several similarity/dissimilarity measures in the same pair of OTUs were considered as a single link. Four networks, one per sampling point, were constructed. Before network construction, samples were filtered such that OTUs were present only in all replicates of the same sampling point; the most abundant OTUs were included. The sum of the filtered OTUs was kept preserving taxon proportions. Then, networks were computed with the 1000 initial top- and bottom-scoring links for each measure. Statistical significance was tested by obtaining the link- and measure-specific *p*-value as the mean of the permutation distribution under the bootstrap distribution, using 1000 iterations for each distribution [32]. Unstable links with scores not within the 95% confidence interval of the bootstrap distribution (e.g., outliers) were removed. *p*-values of different similarity/dissimilarity measures supporting the same link were merged using Brown’s method and corrected for multiple testing using Benjamini–Hochberg’s procedure [51,52]. Finally, networks were filtered to keep only links with an adjusted merged *p*-value below 0.05.

The architecture of each network was portrayed as an undirected, weighted network of microbes’ spatial co-occurrence. The valued matrices (and, if needed, their binary version) were analyzed and visualized using the network analysis (Cytoscape 3.9.1 and UCINET 6 software; [53]). Specifically, we assessed network cohesion, the average geodesic distance among nodes, modularity, small worldness, centrality, network robustness, and the interplay between stochasticity and determinism in the network’s ties (Table S1). Networks’ modularity was assessed by means of the optimization algorithm of Girvan–Newman Q. The values of the optimal partition index (Q) measure the density of ties inside modules compared to ties between modules [54]. An architecture is considered modular if the modularity coefficient Q is greater than 0.4 [55].

A real network is characterized as ‘Small-world’ if its shortest path length (L_real_) is equal to or close to the estimated length of a random counterpart (L) and its clustering coefficient (CI_real_) is significantly higher than its random counterpart (CI_null_). The coefficient of small-worldness (S) is calculated as the ratio of (CI_real_/CI_null_) to (L_real_/L_null_), and a value of S higher than 1 classifies the network as small world. Accordingly, values of the ratio S (coefficient of Small-Worldness) = (Cl_real_/Cl_null_)/(L_real_/L_null_) higher than 1 classify the network to the class of small-world networks [56]. The respective outputs of the experimental networks were tested against corresponding estimates from 1000 Erdös–Rényi random networks using the one-sample *t*-test.

Networks’ robustness to disturbances was evaluated using random graph theory [57]. To do so, we employed the index $p_{s}^{r}$ formulated on the hypothesis that a giant component (i.e., a module comprising more than 50% of the nodes) exists when the average sum of the squares of the degrees is larger than twice the average degree of the network (*K*^2^/*K* > 2). The index, the $p_{s}^{r}$ measures the critical fraction of node removal that causes the network to disintegrate:

$$p_{s}^{r}=1-\frac{1}{\left(K_{0}-1\right)}$$

where $K_{0}=\frac{K^{2}}{K}$, *K* is the average degree of the network, and *K*^2^ is the average of the square of degrees. The larger the positive value of $p_{s}^{r}$, the more robust the network is, whereas the negative values pertain to disintegrated networks.

Another class of alternative indices with an enhanced ability to distinguish between networks is based on considering the eigenvalues of the Laplacian and/or adjacency matrices [58]. In this paper, the natural connectivity of the networks was calculated as follows:

$$\bar{\lambda }=ln\left(\frac{1}{N}\sum_{1}^{N}e^{\lambda_{\iota }}\right)$$

where λ*_i_* is the *i*th eigenvalue of the adjacency matrix. Τhe larger the value of $\bar{\lambda }$, the more robust the network.

To assess the interplay between stochasticity and determinism in the network’s ties the effective information index (*EI*) was estimated according to Klein et al. [58]. First, the tie strengths of each variable *i* were averaged separately for each one, and the Shannon entropy (uncertainty) associated with averages were calculated (determinism component; $H(\bar{W_{i}})$). Then, the Shannon entropy (uncertainty) was calculated separately for each variable, and the individual Shannon indices were averaged (stochasticity component; $\bar{H\left(W_{i}\right)}$. *EI* was the difference between $H(\bar{W_{i}})$ and $\bar{H\left(W_{i}\right)}$:

$$EI=H(\bar{W_{i}})-\bar{H\left(W_{i}\right)}$$

where *H* is the Shannon diversity and *W_i_* is the strength of the node *i*. Finally, *EI* was network-size-normalized as follows:

$$Efectiveness=EI/{log}_{2}\left(N\right)$$

where *N* is the network size, positive values indicate the prevalence of stochasticity, whereas negative values indicate the prevalence of determinism.

## 3. Results

The similarity between bacterial communities’ genera and species was tested by the Bray–Curtis index (Table 1). As regards the overall bacterial community, high compositional similarity (0.68) was recorded between the burned rhizosphere and the unburned bulk soils. The similarity was higher for *Actinomycetes* (1) and lower for *Alphaproteobacteria* (0.31).

**Figure 1:**
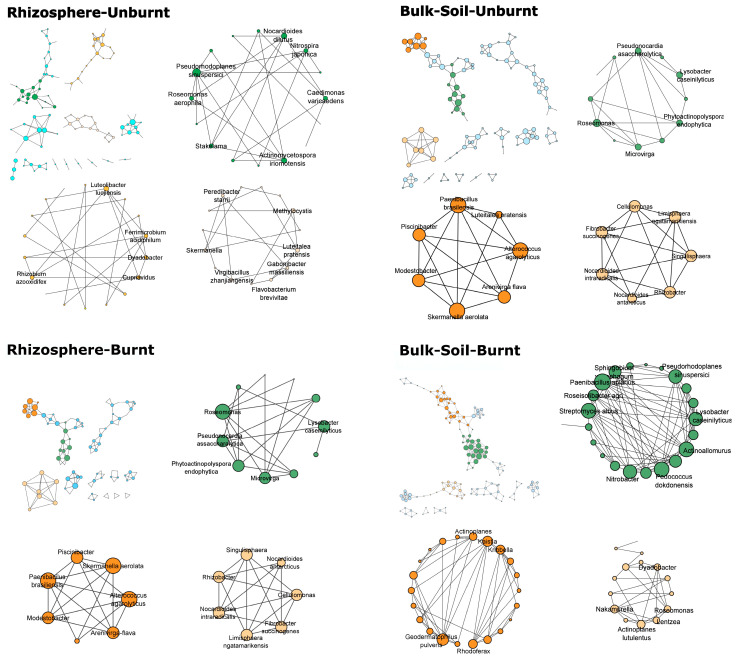
Co-occurrence networks among bacterial taxa sampled in the rhizosphere and bulk soil in burnt and unburnt soils. The size of nodes corresponds to their degree of centrality, and different colors correspond to different modules. In each plot type, the outline of the overall network is presented at the left upper corner, followed by the three biggest modules as these are revealed by the Girvan–Newman algorithm.

As can be realized from Figure 1 in all plots, the network architecture appears as a loose, complex mosaic with a high number of triadic connections, especially in the unburnt rhizosphere network.

Based on the number of nodes, the network was of larger size in the bulk soil of burned plots and smaller in the rhizosphere network of burned plots (Table 2). The rhizosphere network in the unburnt plots had the smallest number of interconnections, the smallest size of the neighborhood, and the lowest centralization but the highest density. The CV values showed low heterogeneity for the size of the neighborhoods, while the density showed increased heterogeneity, except for the network in the rhizospheres of the unburnt plot. The percent centrality of the networks was very low, varying between 1.50 and 5.88, which indicated an even distribution of nodes’ relationships and a lack of hubs. The unburnt rhizospheres’ network showed the highest fragmentation (Table 2). All four networks exhibited modular architecture with corresponding index values above the threshold of 0.4 [54]. A metacommunity structure was depicted, consisting of several modules with strong internal but weak external interconnections. The number of individual nodules ranged from 14 to 21. Finally, the values of the small-world index were higher than unity, indicating modular architecture with small-world properties, i.e., a strong clustering tendency and a prevalence of direct relationships (Table 1).

Table 2 presents values for robustness estimated employing connectivity, with the lowest value found in the unburnt rhizosphere network and the highest in the burned bulk soil—the other two networks had intermediate values. The Eigen centrality supplementary index, which depicts the tendency of strong nodes to connect with other strong ones, showed an amplified range of the above differences (Table 2). Finally, effectiveness values for the three networks were above 0.6, indicating a strong predominance of random processes in the architecture of relationships, while the unburnt rhizosphere network exhibited the lowest effectiveness value, indicating a relatively increased contribution of deterministic processes to its structure.

Based on their degree of centrality, the most influential nodes were categorized into putative functional guilds (biofertilizers, bioprotectants, and decomposers; Table 3). The participation of decomposers was higher in the rhizospheres compared to bulk soils, while the participation of biofertilizers followed the opposite trend. For decomposers and biofertilizers, no effect of fire was recorded. On the other hand, the bioprotectants seem to be affected by fire and spatial scale; their participation was highest in the bulk soils of the burnt plots. The five most influential taxa in the unburnt rhizosphere network belonged completely or partially to the category of biofertilizers, such as *Pseudorhoplanes* [59] and *Hippea* [60], two to the decomposers, *Piscinibacter aquaticus* [61], and *Adhaeribacter* [62], and one to bioprotectants, *Geodermatophilus* [63] (Figure 1). The burnt rhizosphere network had recorded three biofertilizers *Skermanella* [64], *Roseomonas* [65] and *Paenibacillus brasilliensis* [66], one decomposer *Alterococcus* [67], and one bioprotectant *Umezawaea* [68]. Biofertilizers also exert a great influence on the burnt bulk plots where *Roseomonas*, *Skermanella*, *Paenibacillus,* as well as the bioprotectant *Umezawaea* and the decomposer *Alterococcus,* were recorded.

## 4. Discussion

### 4.1. Differences in Network Parameters between Burnt and Unburnt Soil Networks

The values of network parameters varied between sampling points (burnt rhizosphere, unburnt rhizosphere, burnt bulk, unburnt bulk) although not greatly, (except for the robustness index). The larger differences in network parameters were recorded between the unburnt rhizosphere and the burnt bulk soil. Burnt rhizosphere and unburnt bulk soil values were similar and intermediate. The network in the burnt bulk soil was the most compact and centralized, with the highest number of nodes, ties, average neighborhood size, and % degree centralization compared to the other networks. In contrast, the unburnt rhizosphere network had the lowest values of the calculated parameters (except for the number of nodes), resulting in a less connected network lacking central nodes. Apparently, the differences between network parameters cannot be attributed exclusively to fire, as the lethal effect of fire on soil microorganisms is limited since the temperature increase caused by low-severity fires in the first few centimeters of Mediterranean soils is generally low [69], and the post-fire period in the current study was relatively large (33 months). Nevertheless, post-fire conditions have important indirect effects, such as changes in the plant community composition, soil pH, soil chemistry, amount of organic matter, and bulk density [11,20], creating new niches for soil microbes. According to Aponte et al. [27], no differences in the physicochemical soil properties except ΝO_3_^−^ and ΝH_4_^+^ were recorded between burnt and unburnt plots in Pumanque. The differences revealed by the network analysis in bacterial co-occurrence patterns are thus more likely due to the heterogeneity of habitats and niches emerging at the microenvironmental level around the herb root system, partly due to pyrolyzed organic matter (charcoal) that was inserted into the soil [12,13]. The labile components of charcoal are immediately released in burnt plots, while its slow decomposition provides a source of nutrients for a long period of time. It is claimed that the charcoal in the soil creates habitat spots and new niches for microbial colonization. Su et al. [14] found in a burned boreal forest different abundant OTUs and significantly different microbial community compositions on charcoal pieces than in unburned soil. We suggest that the pieces of charcoal and the different physiochemical and biological properties that prevail in the charosphere create a specific habitat whose colonization requires specific skills by microbes.

The incorporation of charcoal into burnt soil increases the structural and functional complexity of the soil and consequently is expected to modify positively or negatively the complexity of the relationships between soil microorganisms. Furthermore, the release of root exudates, which causes significant changes in the carbon and nitrogen cycles, alters the competitive balance between microbes [13]. These perspectives can help explain the differences in network parameters between the two most diversified networks, namely the unburnt rhizosphere and the burned bulk soil network (Table 4 also contains the summarized information per case). Other factors, such as variations in microclimatic variables and resource availability, may also contribute to network differences. For instance, in comparison with resprouting post-fire vegetation in burnt plots, the woody vegetation that prevailed in the unburnt plots could have moderated climate variability. Unburnt soil also has a higher supply of soil resources due to the accumulation of old and fresh litter of various origins and ages (Table 4). In unburnt plots, resources are only needed for maintaining existing biomass, while in burnt plots, they cover the increased needs of fast-growing plants [70]. This results in weaker competition between vegetation and soil microbes in unburnt plots compared to burnt plots. Bonanomi et al. [71] noted strong competition between microorganisms after burning due to nutrient limitation, resulting in a higher number of negative correlations in microbial co-occurrence patterns. The above differences between burned bulk soil and unburned rhizosphere were also reflected in the composition of the key species (nodes with the highest degree of centrality).

Concerning burnt rhizospheres, we assumed enhanced heterogeneity which could be attributed partly to a fresh litter of variable provenance, partly to the addition of habitats owing to charcoal and partly to the increased activity of the fast-regenerating and fire-adapted herbaceous plants [7,31]. The herbaceous assemblages are composed of a variety of resprouting species, each releasing its own root exudates, thereby cultivating its own soil microbial community [3,72]. The root exudates act as a selective factor shaping locally specific and relatively small-sized microbial subnets [4].

Despite habitat differences between burnt and unburnt plots, the configuration of relationships between bacteria in the rhizospheres of the burned soils and in the bulk soil of the unburned areas showed similarities regarding both the community composition of *Actinomycetes* (similarity 100%) and *Alphaproteobacteria* (similarity 31%) and network metrics. Actually, a large amount of litter of variable provenance and age has accumulated on the surface of the unburnt bulk soils, whereas on the surface of the burned rhizosphere, the amount of litter was less and less diversified. Furthermore, the charcoal in the rhizospheres of burned soils likely sequestered a part of root exudates [13], limiting their availability and mediating their role in microbial community composition. Moreover, charcoal could absorb soil organic C, reducing access of microbial communities to labile C. The above, in combination with the lower rate of nitrification reported by Aponte et al. [27], permit us to conclude that a resource regime like that in unburnt bulk soil has been established in the rhizospheres of the burned soils. In sum, the results of our analysis support the first hypothesis, arguing that the accumulated dead material and charcoal produced by fire are key in shaping network architecture and probably explain the similarity between the community composition sampled in the burnt rhizospheres and the unburnt bulk soil (the similarity of the most influential bacterial taxa in the respective communities was 40%), as well as the similarity in the topological network metrics. 

### 4.2. Regulation of Bacterial Communities in Mediterranean Soil Ecosystems: Stochasticity vs. Determinism

Ferrenberg et al. [73] proposed a three-phase conceptual model that describes a phase-dependent contribution of neutral and niche-based processes that shape community structure. This study partially supported this model, showing the highest values of the effectiveness index in burned bulk soil 33 months after the fire, apparently due to the unrestricted dispersal of microbes through wind and/or precipitation, wide fluctuations of climatic variables, and the uneven distribution of charcoal. Relatively smaller stochasticity occurred in the community of burned rhizosphere soils, probably because of root exudates that selected specific microbial subnets from the overall soil microbial pool. Compared to burned areas, the lowest stochasticity (0.388) was recorded in the communities of unburned rhizosphere soils, which could also be attributed to the increased availability of resources such as N forms [33]. According to Liang et al. [74], N addition decreased stochasticity, while Qin et al. [33] found that changes in dissolved organic C regulated the balance between neutral and niche-based processes. In short, these findings are supportive of our second and third hypotheses. In the context of the above suggestions, the burned areas could be assigned to the early stages of a secondary post-fire succession process, while that of the unburned rhizosphere fits well with the second phase of succession where habitat filtering prevails. An exception from the model was the community in unburned bulk soil that followed assembly rules, such as those of burned areas. Our analysis was applied to taxa that were present in all replicates of each sampling point exhibiting wider niche width. These findings align with Qin et al. [33], who found that, compared to unburned soil, in burned soils, stronger stochastic processes were associated with abundant bacteria and suggested that abundant taxa follow strategies related to nutrient acquisition compared to rare taxa whose strategies related to avoidance or tolerance to the stress imposed by fire.

The balance between stochasticity and determinism in regulating bacterial communities can also be discussed in terms of modularity and small-world properties that are considered important in terms of network robustness and stability [75]. Our results revealed modular networks with small-world properties, indicating that stochastic processes were dominant, leading to uniform local architectures [35] and the easy diffusion of secondary metabolites throughout each modulus/community [39]. Moreover, modular architecture endowed with small-world properties induces time-scale separation of processes at the metacommunity level, in that processes within modules/local communities proceed quickly, while those between modules/local communities proceed slowly [76]. In such networks, local repairs due to the free movement of recolonizing microbes make the modular metacommunity resistant and resilient against global disturbance [41]. In this paper, the robustness of the soil bacterial metacommunities appeared enhanced in burnt plots and was more pronounced in bulk soil (5.3 vs. 1.95 connectivity, respectively; Table 2). We suggest that this is attributable to the long evolutionary history of Mediterranean ecosystems, where fire is tightly connected with their dynamics.

The metacommunity architecture, which was common for all networks, seems to fit well with the diversified mosaic of shrubby and herbaceous vegetation, voids, stones, and outcropping rocks of the Mediterranean habitats [1], but whether this is a typical feature of the Mediterranean-type ecological networks remains to be confirmed in other studies. Our results were similar to Bonanomi et al. [71], who found in a 5-year experiment that the microbial networks in burnt and mowed soils formed many dispersed subcommunities. Fragmented networks are also reported for other flammable ecological formations, such as boreal forests [14,77], where the complexity and connectivity of bacterial and fungal communities significantly increased 17 years after a fire, compared with either unburnt soils or soils with recent fires.

As aforementioned, it is argued that metacommunity architecture leads to increased resilience of the microbial communities against disturbance. Following the approach of Mayfield and Levine [40], we posit that the conditions in burnt soils may facilitate recolonization not only through stochastic unlimited dispersal of microbes with varying niches but also through differences in their competitive ability. Specifically, competitive ability due to niche differences contributes to the observed spatial pattern characterized by over-dispersed modules and small-world properties through which hazards, as well as rapid healing processes of damage repair, are shared across space and time, probably enhancing the resilience of the Mediterranean microbial metacommunity to repeated disturbances such as overgrazing and fire. 

## 5. Conclusions

We concluded that the architectural features (modularity and small-world properties) found in all four networks were likely related to the adversities of the Mediterranean environments and their mosaic spatial pattern. Fire similarly affected some architectural features in either the rhizosphere or the bulk soil. The networks in the burnt areas contained more co-occurring taxa, were less fragmented, had lower null ties, exhibited stronger local organization, and had larger neighborhoods. Thus, at first glance, they were more complex and met all the criteria to be considered more stable than the networks in unburnt areas. However, the robustness of the networks was the product of the joint effect of fire and the small spatial arrangement of samples; fire increased robustness, but this was more pronounced in burnt bulk soil. The mechanisms mainly responsible for bacterial community structure were stochastic in all soils, either burnt or unburnt, a fact that contrasts with one of our initial hypotheses. The contribution of stochasticity changed in rhizosphere soils; networks in burnt rhizospheres were much more stochastic than in unburnt rhizospheres. Mechanisms controlled stochasticity related to the abundance and quality of litter, the variability in microclimatic variables, the presence/absence of charcoal, the unlimited dispersal, and the intrinsic environmental heterogeneity of the Mediterranean soils. Overall, our study provides valuable insights into the complexity and robustness of bacterial networks in burnt Mediterranean soils; however, distinguishing the fire effect from the rhizosphere effect is difficult and highlights the need for further research to disentangle the effects of these two agents on bacterial community structure.

## Figures and Tables

**Table 1:** Bray–Curtis index of similarity applied in the overall bacterial community and the community of *Actinomycetes* and *Alphaproteobacteria* collected in the different plots.

		Rhizosphere		Bulk Soil	
		Unburnt	Burnt	Unburnt	Burnt
		Total Bacteria			
Rhizosphere	Unburnt	1	0.09	0.09	0.16
	Burnt		1	0.68	0.13
Bulk soil	Unburnt			1	0.13
	Burnt				1.00
		*Actinomycetes*			
Rhizosphere	Unburnt	1	0	0	0.32
	Burnt		1	1	0
Bulk soil	Unburnt			1	0
	Burnt				1
		*Alphaproteobacteria*			
Rhizosphere	Unburnt	1	0.08	0	0.23
	Burnt		1	0.31	0
Bulk soil	Unburnt			1	0
	Burnt				1

**Table 2:** Co-occurrence network properties for networks corresponding to each sampling site. For the description of parameters, see Table S1 (STDEV = standard deviation).

	Bulk Burnt (BB)	Bulk Unburnt (BU)	Rhizosphere Burnt (RB)	Rhizosphere Unburnt (RU)
Nb. of nodes	130	108	93	125
Νb. of ties	590	344	318	154
Avg Νeighborhood size (ANS)	4.538	3.185	3.419	2.933
ANS (STDEV)	2.37	1.348	1.247	1.389
% Deg Centralization	5.875	2.655	2.835	1.500
Density	0.035	0.030	0.037	0.101
Density (STDEV)	0.184	0.170	0.189	0.046
Fragmentation	0.708	0.858	0.839	0.870
Modularity	0.814	0.861	0.868	0.861
Nb. Modules	17	20	14	21
Avg Distance	5.289	3.892	3.583	4.644
Compactness	0.095	0.060	0.072	0.050
Clustering Coefficient	0.623	0.593	0.649	0.239
Small Worldness	10.346	20.097	16.674	12.985
Nulls	0.965	0.970	0.962	0.980
Robustness-Critical faction	0.791	0.637	0.652	0.554
Robustness-Natural Connectivity	5.300	1.954	2.995	2.000
Effectiveness	0.691	0.624	0.648	0.388

**Table 3:** Classification of bacterial taxa to putative functional groups.

	Rhizosphere		Bulk Soil	
	Unburnt (RU)	Burnt (RB)	Unburnt (BU)	Burnt (BB)
Nb. of influential nodes	19	19	21	22
% Biofertilizers	37	37	40	41
% Bioprotectants	22	11	19	27
% Decomposers	42	53	33	32

**Table 4:** Processes and agents affecting bacterial co-occurrence patterns in the burnt and unburnt rhizosphere and bulk soils.

	Rhizosphere	Bulk
Unburnt	Vegetation of high taxonomic diversity Abundant litter of different ages and quality (more diversified) High diversity of root exudates Plenty of resources Weak competition between plants and microbes Less contribution of stochasticity to microbial community assembly	Vegetation of high taxonomic diversity Abundant litter of different ages and quality (more diversified) Shortage of root exudates Limitation in resources Intermediate contribution of stochasticity to microbial community assembly
Burnt	Vegetation of low taxonomic diversity exhibiting high growth rates and/or biomass Litter of specific age, of lower amount and less diversified Low diversity of root exudates Charcoal presence leads to new niches and increased heterogeneity for soil microbes Charcoal presence binds root exudates Limitation in resources Strong competition between plants and microbes due to limitations imposed by fire and charcoal presence Intermediate contribution of stochasticity to microbial community assembly	Vegetation of low taxonomic diversity exhibiting high growth rates and/or biomass Litter of specific age, of lower amount and less diversified Shortage of root exudates Charcoal presence leads to new niches and increased heterogeneity for soil microbes The highest limitation in resources due to low plant diversity, fire, charcoal presence, shortage of exudates Highest heterogeneity for soil microbes Large contribution of stochasticity to microbial community assembly

## Data Availability

The data sets analyzed in this study were originated by Aponte et al. (2022) and are available in the Sequence Read Archive (SRA) of the National Center for Biotechnology Information (NCBI) under accession number PRJNA784510.

## Supplementary Materials

The following supporting information can be downloaded at: https://www.mdpi.com/article/10.3390/microorganisms11030790/s1, Table S1: Description of the studied network parameters.
