# Supplementary material for: Fire and Rhizosphere Effects on Bacterial Co-Occurrence Patterns
Source: Microorganisms. 2023 Mar 19;11(3):790. doi: 10.3390/microorganisms11030790 (PMC10052084; doi:10.3390/microorganisms11030790)
Supplement: Supplementary file 1 [file microorganisms-11-00790-s001.zip › microorganisms-2234427-supplementary.pdf]

Table S1. Description of the studied network parameters

	Avg Neighborhood size	The number of nodes (alters) that a node is directly connected to.
Cohesion	Density	For a binary network it is equal to the total number of ties divided by the total number of possible ties.<br><br>For a valued network it is equal to the total of all values divided by the number of possible ties.
	Fragmentation	Proportion of node pairs that are unreachable
	Compactness	Returns the probability that two random nodes will be connected to each other.
	Nulls	Proportion of pairs of nodes that are not connected to each other.
	Geodesic distance	The geodesic distance between two nodes is the number of ties between nodes n_i and n_j.
Centrality	Degree	The degree of each node is equal to the sums of the weights (correlations) of its links with other nodes. It calculates how central this node is in the network, thus measuring the size of its influence.
Clustering	Modularity	Split of nodes into distinct modules (subnets), wherein the number of internal ties of each modulus is greater than the number of ties with other nodules. It accounts for the tendency of nodes to establish direct connections. Modular architectures are considered of increased stability.
	Clustering coefficient	The clustering coefficient of a node is the average weight of ties among the nodes directly connected with it (its open neighborhood). The overall clustering coefficient is the mean of the clustering coefficients of all the nodes
Small world	Small-world properties	Small high intra-cluster and reduced inter-cluster connectivity between nodes.
	Small world architecture	The distribution of connections at the network-wide level is random, but at the local subnet level the distribution is regular.
	Small-world coefficient	A real network is small world if it has a similar mean shortest path length, but greater clustering coefficient compared to corresponding random network

Resistibility	Robustness	The ability of a network to maintain its connectivity in response to disturbances. Robustness is in a conflicting relation with the Small-World effect
	Effectiveness	Accounts for the interplay between stochasticity (degeneracy) and determinism in network’s ties
